# The Manchester Procedure as a Uterine-Preserving Alternative for Uterine Prolapse Due to Cervical Elongation: A Short- and Mid-Term Clinical Analysis

**DOI:** 10.3390/medicina61071183

**Published:** 2025-06-29

**Authors:** Claudia Liger Guerra, Lorena Sabonet Morente, Juan Manuel Hidalgo Fernandez, Manuel Navarro Romero, Cristina Espada Gonzalez, Jesus S. Jimenez-Lopez

**Affiliations:** 1 Department of Surgical Specialties, University of Malaga, 29010 Málaga, Spain; claudialigierguerra@gmail.com; 2 Obstetrics and Gynecology Department, Hospital Materno-Infantil, Hospital Regional Universitario Málaga, Avenue Arroyo de los Ángeles S/N, 29011 Málaga, Spain; lorenasabonet@gmail.com (L.S.M.); jmgine@gmail.com (J.M.H.F.); navarroromero.m@gmail.com (M.N.R.); cristinaespadag@hotmail.com (C.E.G.); 3 Research Group in Maternal-Fetal Medicine Epigenetics Women’s Diseases and Reproductive Health, Biomedical Research Institute of Malaga (IBIMA), 29071 Málaga, Spain

**Keywords:** pelvic organ prolapse, Manchester procedure, cervical elongation, uterine preservation, urogynecology, patient satisfaction, surgical outcomes

## Abstract

*Background and Objectives:* Pelvic organ prolapse (POP) is a prevalent condition that negatively impacts women’s quality of life. Uterine-preserving procedures are increasingly demanded by patients with uterine prolapse, particularly when associated with true cervical elongation. The Manchester procedure, historically used for uterine preservation, has regained interest due to its effectiveness and low morbidity. This study aims to evaluate the anatomical and functional outcomes of the Manchester procedure in women with uterine prolapse due to cervical elongation, assessing patient satisfaction and associated clinical factors. *Materials and Methods:* We conducted a retrospective, observational, single-center study at the Regional University Hospital of Málaga, Spain, including patients undergoing the Manchester procedure between January 2017 and December 2022. Inclusion criteria required a diagnosis of uterine prolapse due to clinically confirmed true cervical elongation. Surgical details, complications, and postoperative outcomes were recorded. Patient satisfaction was assessed using a Likert scale during follow-up visits. *Results:* A total of 38 patients were included, with a mean age of 48.7 years. All presented with symptomatic uterine prolapse and elongated cervix (>5 cm). The anatomical success rate was 97%, with only one case of symptomatic recurrence. The most common early postoperative complication was urinary tract infection (10.5%). The average follow-up duration was 18.6 months. A high level of satisfaction was recorded: 94.8% of patients were either “very satisfied” (73.7%) or “satisfied” (21.1%), and only 5.3% reported dissatisfaction. Multicompartmental repair (anterior and/or posterior colporrhaphy) improved satisfaction outcomes. *Conclusions:* The Manchester procedure is a safe, effective uterine-sparing surgical option for patients with cervical elongation-related uterine prolapse. It demonstrates a high anatomical success rate and low morbidity, with excellent patient satisfaction. Comprehensive preoperative assessment and addressing modifiable risk factors such as obesity and smoking are key to optimizing results. Further prospective studies are needed to assess long-term durability and quality-of-life outcomes.

## 1. Introduction

Pelvic organ prolapse (POP) is a highly prevalent and progressively disabling condition that significantly impairs the quality of life of millions of women worldwide. It is characterized by the descent of pelvic structures—primarily the uterus, bladder, or rectum—into or through the vaginal canal due to weakening of the pelvic floor support system. The prevalence of POP increases with age, parity, and specific comorbidities, with epidemiological data indicating rates of up to 14.2% among postmenopausal women. However, POP is not exclusive to this population and may also affect younger and nulliparous women, especially in the presence of connective tissue disorders or a familial predisposition.

Among the different clinical presentations of POP, uterine prolapse associated with true cervical elongation represents a distinct anatomical and therapeutic challenge. In this condition, the uterus descends primarily as a result of pathological cervical elongation, while the uterine corpus remains adequately supported. Accurate diagnosis—through both physical examination and imaging studies such as transvaginal ultrasound—is essential to distinguish it from other forms of uterine descent and to guide appropriate surgical management.

Historically, vaginal hysterectomy has been the most common surgical approach for advanced uterine prolapse. However, the paradigm has shifted in recent decades, as increasing numbers of women express a desire to preserve the uterus—not only for future fertility but also due to psychosocial and emotional reasons. Qualitative studies have shown that uterine preservation can positively influence body image, female identity, and sexual function.

As a result, uterine-sparing techniques have gained renewed attention, particularly the Manchester procedure. Originally introduced by Archibald Donald in 1888 and later refined by Thomas Fothergill, this surgery involves subtotal cervical amputation, the plication of the cardinal and uterosacral ligaments to the cervical stump, and the restoration of apical support. Initially developed during the Industrial Revolution to treat multiparous, working-class women, the procedure aimed to preserve reproductive capacity at a time when childbearing was essential.

Although largely replaced by hysterectomy-based techniques during the 20th century due to advancements in anesthesia and surgical safety, the Manchester procedure has regained clinical relevance. Recent evidence supports its effectiveness, demonstrating outcomes comparable or even superior to more invasive alternatives in terms of anatomical correction, lower intraoperative morbidity, shorter operative time, and fertility preservation.

Comparative studies have shown that the Manchester procedure results in lower intraoperative blood loss, shorter hospital stays, and equal or improved functional outcomes compared to vaginal hysterectomy or sacrospinous hysteropexy. One randomized trial reported a 2-year surgical success rate of 89.1% for the Manchester procedure, compared to 78.5% for sacrospinous hysteropexy. Moreover, in young women with reproductive goals, the Manchester technique has shown favorable fertility outcomes, with post-surgical pregnancy rates of up to 43%, although some obstetric risks remain.

Despite these promising outcomes, clinical debate continues. Long-term durability, particularly regarding recurrence in other pelvic compartments (anterior or posterior), remains uncertain. Additionally, there are concerns regarding oncological surveillance, specifically cervical cancer screening, in women with preserved uterine tissue. The lack of standardized protocols and variability in patient selection criteria further complicate the generalization of results across populations.

Given these considerations, further clinical research is necessary to assess the Manchester procedure in real-world settings, especially with respect to short- and mid-term anatomical and functional outcomes, patient satisfaction, and complication profiles.

The main objective is to evaluate the short- and mid-term clinical outcomes of the Manchester procedure in patients with uterine prolapse secondary to true cervical elongation.

The specific objectives are to analyze the anatomical and functional success of the procedure in terms of prolapse correction and the resolution or persistence of symptoms, to assess patient satisfaction following surgery and identify factors associated with complete, partial, or absent satisfaction, to determine the incidence and nature of intraoperative and postoperative complications, and to explore associations between clinical variables (e.g., BMI, parity, smoking, comorbidities) and surgical outcomes.

## 2. Materials and Methods

### 2.1. The Study Design

This observational, retrospective, and analytical study was conducted at the Regional University Hospital of Málaga, Spain. A review of medical records was performed for patients who underwent the Manchester procedure between January 2017 and December 2022.

### 2.2. Study Population

All patients diagnosed with uterine prolapse secondary to true cervical elongation were included. The diagnosis was clinically confirmed through the bimanual pelvic examination and direct visualization of the cervix. True cervical elongation was defined as a cervical length of 5 cm or greater, measured from the external cervical to the top of the cervix, in the absence of significant uterine corpus descent. The study was approved by the Institutional Research Ethics Committee. This study was conducted in accordance with the Declaration of Helsinki and approved by the Ethics Committee of the CEI de Provincial 168 Centre of Málaga, Spain (protocol code 2025-001035 and date of approval 20 April 2025).

Patients were excluded if they presented with uterine prolapse associated with primary apical support deficiency (i.e., weakness of the uterosacral or cardinal ligaments without cervical elongation) or suspected or confirmed premalignant or malignant cervical pathology or if they did not complete a minimum postoperative follow-up period of six months.

### 2.3. Surgical Procedure

The Manchester procedure was performed by gynecologic surgeons specialized in pelvic floor reconstructive surgery, under regional or general anesthesia. The technique consisted of the following steps:

1. Subtotal amputation of the cervix;
2. Plication of the uterosacral and cardinal ligaments anterior to the cervical stump using non-absorbable sutures;
3. Reconstruction of the cervical canal and closure of the stump using a modified Sturmdorf technique;
4. Concomitant anterior and/or posterior colporrhaphy when indicated, based on the intraoperative identification of relevant defects.

The decision to perform additional anterior or posterior colporrhaphy was made on a case-by-case basis, depending on the presence of significant cystocele or rectocele.

### 2.4. Study Variables

The following variables were collected:

- **Sociodemographic data:** age, parity, menopausal status, body mass index (BMI), smoking status, and comorbidities (diabetes mellitus, hypertension);
- **Clinical characteristics:** degree of prolapse based on the POP-Q classification, associated urinary or bowel symptoms, and presence of sexual dysfunction;
- **Intraoperative data:** operative time, estimated blood loss, need for transfusion, intraoperative complications;
- **Postoperative outcomes:** pain, vaginal bleeding, fever, urinary tract infection, urinary retention, wound dehiscence, and reinterventions;
- **Anatomical and functional evaluation:** recurrence of prolapse, persistence or emergence of new symptoms, and overall patient satisfaction, assessed using a Likert-type scale (very satisfied, satisfied, or dissatisfied);
- **Presence of sexual dysfunction:** sexual dysfunction was assessed based on spontaneous patient reports during clinical follow-up; however, no validated questionnaire (e.g., PISQ-12) was employed. This represents a limitation in the standardized assessment of this outcome.

### 2.5. Follow-Up

Patients were evaluated during follow-up visits at 6 weeks, 6 months, and 12 months postoperatively, and annually thereafter. Follow-up assessments included pelvic examination, the evaluation of anterior, apical, and posterior compartments using the POP-Q system, and patient-reported outcomes regarding symptom improvement and quality of life.

### 2.6. Statistical Analysis

Data were analyzed using SPSS software version 25.0. Measures of central tendency and dispersion were used for continuous variables, and absolute and relative frequencies were used for categorical variables. Associations were analyzed using the chi-square test, Student’s *t*-test, or Mann–Whitney U test, as appropriate. A *p*-value of <0.05 was considered statistically significant.

## 3. Results

During the period from January 2017 to December 2022, a total of 38 patients underwent the Manchester procedure as surgical treatment for uterine prolapse secondary to true cervical elongation. The sociodemographic and clinical characteristics of this cohort are summarized in Table 1.

The mean age of the patients was 48.7 ± 6.4 years (range: 37–62 years), with an average parity of 3.9 ± 1.1 deliveries. Regarding hormonal status, 63.2% of the patients (n = 24) were postmenopausal at the time of surgery. The mean body mass index (BMI) was 27.3 ± 2.5 kg/m^2^.

The most common comorbidities were hypertension (39.5%, n = 15), type 2 diabetes mellitus (21.1%, n = 8), and active smoking (13.2%, n = 5). Preoperative clinical evaluation using the POP-Q classification revealed that 28.9% of patients were at stage II, 65.8% at stage III, and 5.3% at stage IV.

With regard to presenting symptoms, 100% of the patients reported a sensation of vaginal bulge as the main reason for consultation. In addition, stress urinary incontinence was documented in 23.7% of cases (n = 9), sexual dysfunction in 34.2% (n = 13), and constipation or difficulty with defecation in 15.8% (n = 6). (Table 1)

Regarding the surgical details of the Manchester procedure, the mean operative time was 78.5 ± 12.3 min, and the mean estimated blood loss was 154.2 ± 30.5 mL. No intraoperative complications were reported in any of the cases, reflecting a favorable safety profile for this technique in the context of true cervical elongation.

In addition to the primary procedure, complementary surgical interventions were performed based on each patient’s individual anatomical findings. Specifically, 50% of patients (n = 19) underwent anterior colporrhaphy, 36.8% (n = 14) required posterior colporrhaphy, and both procedures were performed simultaneously in 18.4% of cases (n = 7). (Table 2)

During early postoperative follow-up (≤30 days), a number of minor complications were observed, all of which were managed conservatively without the need for surgical reintervention. Postoperative fever, defined as a body temperature above 38 °C, was reported in two patients (5.3%). The most frequent complication was a urinary tract infection, diagnosed in four patients (10.5%), followed by transient urinary retention in three cases (7.9%).

Additionally, one patient (2.6%) presented with a hematoma or persistent vaginal bleeding, and another case (2.6%) involved mild dehiscence of the cervical stump, without clinical significance or need for additional surgical treatment (Table 3).

Mid-term clinical follow-up revealed favorable outcomes in the majority of patients. The mean follow-up duration was 18.6 months, ranging from 6 to 48 months. During this period, only one case (2.6%) of symptomatic prolapse recurrence was documented, localized in the anterior compartment and occurring 24 months after the initial procedure.

**Figure 1:**
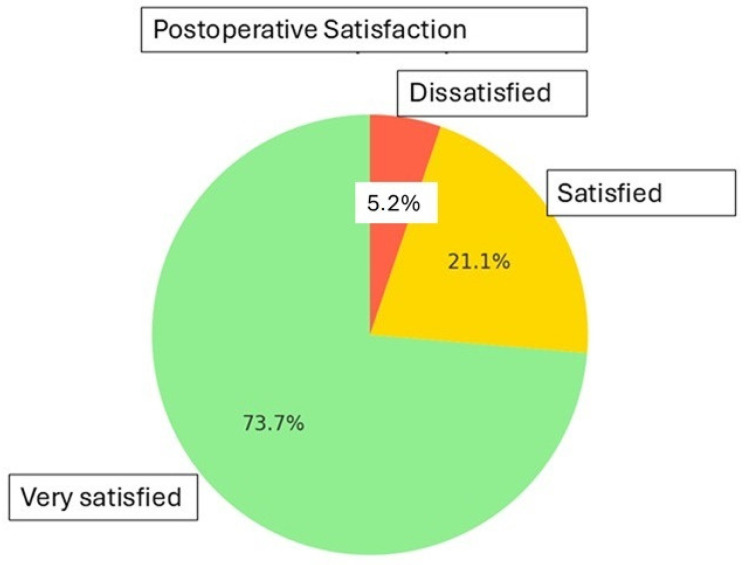
Postoperative satisfaction.

Regarding persistent or new-onset symptoms, two patients (5.3%) developed de novo urgency urinary incontinence and three patients (7.9%) reported persistent dyspareunia. Despite these findings, overall satisfaction with the procedure was high. According to the Likert scale, 73.7% of patients (n = 28) reported being “very satisfied,” 21.1% (n = 8) “satisfied,” and only 5.3% (n = 2) expressed dissatisfaction with the surgical outcome. (Table 4 and Figure 1)

## 4. Discussion

The Manchester procedure has traditionally been considered an effective surgical alternative for the treatment of uterine prolapse in patients who wish to preserve the uterus, particularly when true cervical elongation is the primary component of apical support defect. Our findings support this premise, demonstrating that the technique can yield satisfactory functional outcomes, with a low recurrence rate and minimal complications [1,2,3,4,5].

This study not only provides clinically relevant data in a selected patient population but also addresses the growing demand for less invasive surgical procedures that prioritize uterine preservation. In this context, the Manchester technique represents a valid and safe surgical option, aligned with the values of reproductive autonomy and psychological well-being upheld by many patients. Its implementation may be considered within individualized surgical protocols, provided that appropriate anatomical and functional assessment is ensured.

In our series, most patients were multiparous and postmenopausal—both well-established risk factors for genital prolapse [1,2]. True cervical elongation was documented in all cases through clinical assessment and functional evaluation, and it was the primary criterion for selecting the Manchester procedure over conventional hysterectomy.

Overall functional outcomes were favorable: 94.8% of patients reported being either satisfied or very satisfied with their surgical results, and the symptomatic recurrence rate was low (2.6%), consistent with rates reported in the literature, which range from 2% to 10% depending on the study and follow-up period [3,4]. These results align with those of Fayyad and Sayed, who reported success rates exceeding 89% in patients with cervical elongation treated using this technique [5].

Intraoperative and early postoperative complications were infrequent and mild. No visceral injuries or need for reintervention were reported. The most common complication was a urinary tract infection, observed in 10.5% of patients, which is comparable to the expected incidence for vaginal procedures [5,6]. This favorable safety profile has also been confirmed by recent multicenter studies, which emphasize the low morbidity of the Manchester procedure compared to more invasive techniques such as vaginal hysterectomy or sacrospinous hysteropexy [7,8].

The rate of de novo urgency urinary incontinence observed in our cohort (5.3%) is lower than that reported in other prolapse surgery series. Previous studies have documented rates of up to 15–16% following procedures with or without suburethral sling placement [1,2]. This difference may be attributed to the reduced manipulation of the anterior compartment and the absence of prosthetic materials in the Manchester technique, further supporting its favorable functional profile in terms of urinary morbidity.

Importantly, the Manchester procedure preserves the uterus, which can have a positive psychological impact in selected patients. Although most of our cohort was postmenopausal, the desire to avoid hysterectomy remained a relevant factor in several cases—consistent with recent studies that support uterine-preserving surgery when clinically appropriate [2,3]. Uterine preservation has been shown to improve body image perception, female identity, and sexual function—factors especially important in younger women or those with a history of gynecological trauma [3].

Furthermore, in women of reproductive age, the Manchester procedure has demonstrated postoperative pregnancy rates of up to 43%, reinforcing its utility in patients with future fertility goals [7]. Nonetheless, potential obstetric risks—such as preterm birth or cervical insufficiency—must be discussed in advance with the patient.

Key limitations of our study include its retrospective design and relatively small sample size. Additionally, mid-term follow-up does not allow for definitive conclusions regarding long-term durability. However, it provides valuable evidence supporting a surgical technique that, although less frequently employed today, continues to play an important role in individualized urogynecological care [8]. Recent studies have noted that anterior compartment recurrence may be more frequent following uterine-sparing procedures, underscoring the importance of thorough preoperative multicompartmental assessment [9].

These findings are consistent with recent international guidelines from the IUGA and ICS, which support uterine-preserving techniques—including the Manchester procedure—in appropriately selected patients with cervical elongation. These guidelines emphasize shared decision-making, anatomical assessment, and the psychological and reproductive benefits of uterine conservation [10].

Our findings support the Manchester procedure as a safe, effective, and well-accepted surgical option, particularly in settings where uterine preservation is a clinical or personal priority.

## 5. Conclusions

The Manchester procedure remains a valid, safe, and effective surgical alternative for the treatment of uterine prolapse associated with true cervical elongation, particularly in appropriately selected patients. This study confirms a high anatomical success rate (97%) and minimal intraoperative morbidity, with no major complications.

Overall patient satisfaction was high, with 97.1% of patients reporting positive outcomes: 61% experienced complete resolution of symptoms, while 36.1% reported partial but clinically significant improvement. These findings support both the functional effectiveness and the strong patient acceptance of the procedure.

To optimize outcomes, it is essential to identify and address risk factors such as obesity and smoking and to perform a comprehensive pelvic floor evaluation to repair other involved compartments when indicated. Prospective, multicenter studies with long-term follow-up are recommended to assess durability and quality-of-life outcomes.

## Figures and Tables

**Table 1:** Sociodemographic and clinical characteristics of the patients.

Variable	Value
Mean age (years)	48.7 ± 6.4 (range: 37–62)
Mean parity (number of deliveries)	3.9 ± 1.1
Postmenopausal status	24 patients (63.2%)
Mean BMI (kg/m^2^)	27.3 ± 2.5
Active smoking	5 patients (13.2%)
Hypertension	15 patients (39.5%)
Type 2 diabetes mellitus	8 patients (21.1%)
**Prolapse stage (POP-Q classification)**	**Number of patients (%)**
Stage II	11 patients (28.9%)
Stage III	25 patients (65.8%)
Stage IV	2 patients (5.3%)
**Preoperative clinical symptoms**	**Number of patients (%)**
Vaginal bulge sensation	38 patients (100%)
Stress urinary incontinence	9 patients (23.7%)
Sexual dysfunction	13 patients (34.2%)
Constipation/difficulty with defecation	6 patients (15.8%)

**Table 2:** Surgical details of the Manchester procedure.

Variable	Value
Mean operative time (minutes)	785 ± 123
Mean estimated blood loss (mL)	1542 ± 305
Intraoperative complications	None reported
**Additional surgical procedures**	**Number of patients (%)**
Anterior colporrhaphy	19 patients (50.0%)
Posterior colporrhaphy	14 patients (36.8%)
Both anterior and posterior	7 patients (18.4%)

**Table 3:** Early postoperative complications (≤30 days).

Complication	Number of Patients (%)
Postoperative fever (>38 °C)	2 patients (5.3%)
Urinary tract infection	4 patients (10.5%)
Transient urinary retention	3 patients (7.9%)
Hematoma or persistent vaginal bleeding	1 patient (2.6%)
Mild cervical stump dehiscence	1 patient (2.6%)

**Table 4:** Mid-term outcomes and patient satisfaction.

Variable	Value
Mean follow-up duration (months)	18.6 (range: 6–48)
Symptomatic prolapse recurrence	1 patient (2.6%)—anterior compartment
**Persistent or new symptoms**	
De novo urgency urinary incontinence	2 patients (5.3%)
Persistent dyspareunia	3 patients (7.9%)
**Patient satisfaction (Likert scale)**	
Very satisfied	28 patients (73.7%)
Satisfied	8 patients (21.1%)
Dissatisfied	2 patients (5.2%)

## Data Availability

The original contributions presented in the study are included in the article; further inquiries can be directed to the corresponding author.
